# Are current etiological theories of Alzheimer’s disease falsifiable? An epistemological assessment

**DOI:** 10.3389/fnagi.2025.1708234

**Published:** 2025-11-28

**Authors:** Tommaso Costa, Donato Liloia

**Affiliations:** 1 Functional Neuroimaging and Complex Neural Systems (FOCUS) Laboratory, Department of Psychology, University of Turin, Turin, Italy; 2 Computational Neuroimaging & Complex Systems Group, GCS-fMRI Koelliker Hospital, Turin, Italy; 3 Neuroscience Institute of Turin (NIT), Turin, Italy; 4 Translational Neuroimaging & Brain Connectivity Group, GCS-fMRI Koelliker Hospital, Turin, Italy

**Keywords:** epistemology of science, falsifiability, Bayesian inference, eliminative induction, scientific models, theory comparison, predictive modeling, scientific rationality

## Abstract

Alzheimer’s disease (AD) research is plagued by a proliferation of competing etiological theories, often coexisting without undergoing systematic critical comparison. This article examines the epistemological limitations of the traditional falsifiability criterion, formulated by Karl Popper, and demonstrates how this principle fails to function effectively in the context of AD research. Biological complexity, the absence of unequivocal biomarkers, institutional resistance to paradigm shifts, and academic incentives to preserve dominant hypotheses all contribute to the erosion of falsifiability as an operational standard. In response, we propose an alternative framework based on Bayesian inference, understood as eliminative induction—a process in which scientific theories are modeled as probabilistic hypotheses with gradable plausibility, continuously updated considering new evidence. Within this framework, models are not regarded as literally “true,” but as pragmatic tools whose predictive performance determines their scientific value. We advocate for a more comparative, predictive, and transparent scientific practice, wherein progress does not hinge on identifying a unique cause or on proving (or disproving) a hypothesis, but rather on enhancing our ability to rationally distinguish among competing models using quantitative criteria.

## 1 Introduction

Alzheimer’s disease (AD) is the most prevalent form of dementia, accounting for approximately 60–80% of cases (Knopman et al., 2021). It is a progressive neurodegenerative condition characterized by a gradual decline in cognitive functions, including memory, executive functioning, and language abilities (Breijyeh and Karaman, 2020). The incidence of AD rises markedly with age, particularly beyond 65 years, mirroring global demographic trends of population aging. Among individuals aged 65 and older, AD significantly reduces healthy life expectancy, contributing to profound cognitive, behavioral, and functional impairments. These deficits not only diminish patients’ quality of life but also place a considerable emotional and economic burden on caregivers (Liu et al., 2025).

Despite decades of intensive research and substantial worldwide financial investment, AD remains one of the most elusive and challenging neurological disorders, both clinically and epistemologically. Multiple theories have been proposed to explain its etiology, ranging from the classical amyloid cascade hypothesis (Hardy and Higgins, 1992; Roberts et al., 1993; Selkoe, 2000; Ethell, 2010; Anastasio, 2011; Tam and Pasternak, 2012; Castellani et al., 2019; Paroni et al., 2019; Petrella et al., 2019) to other hypotheses involving the cerebrovascular and blood–brain barrier abnormalities (Kalaria, 1992; de la Torre, 1999; Zlokovic, 2002; Pluta and Amek, 2008; Cordonnier and van der Flier, 2011; Orehek, 2012; Kapadia et al., 2020), inherited genetic mutations (Yurov et al., 2011; Bruni et al., 2020; Kaeser and Chun, 2020), gut–brain axis (Angelucci et al., 2019; Bostanciklioğlu, 2019; Cerovic et al., 2019; Roe, 2022), inflammation/immunological response (Barger, 2004; Bermejo-Pareja et al., 2020; de Oliveira et al., 2021; Bowirrat, 2022; Heneka et al., 2025), mitochondrial dysfunction (Bonilla et al., 1999; Bonda et al., 2009; Swerdlow and Khan, 2009; Chen and Nguyen, 2014; Albensi, 2019; Ebanks et al., 2020), neurotransmitter deficits (Schmitt, 2005; Craig et al., 2011; Bi et al., 2020; Zorec and Vardjan, 2023), oxidative stress (Benzi and Moretti, 1995; Veurink et al., 2003; Aliev et al., 2009; Lloret et al., 2021; Roy et al., 2023), pathogens infection (Dobson and Itzhaki, 1999; Dezfulian et al., 2008; Block, 2019; Fülöp et al., 2020; Li et al., 2021; Sait et al., 2021; Yong et al., 2021), and Tau proteinopathy (Maccioni et al., 2010; Kametani and Hasegawa, 2018; Arnsten et al., 2021; Chen and Yu, 2023). However, it is important to note that none of these theoretical and experimental proposals has succeeded in offering a comprehensive understanding of the disease or in guiding the development of effective, disease-modifying therapies (Hüll et al., 2006; Cummings et al., 2018; Golde, 2022; Belder et al., 2023; Costa et al., 2023; Edwards and Corkill, 2023; Watt et al., 2023; Duchesne et al., 2024).

This proliferation of several theoretical points of view raises a critical epistemological issue: to what extent are current etiological theories of AD actually falsifiable, in accordance with Karl Popper’s criterion of scientific validity?

The concept of falsifiability, introduced by Popper (1959), forms the crux of the debate on the distinction between science and pseudoscience. A theory is deemed scientific only if it is conceivable that observations could refute it. Conversely, if a theory is compatible with every conceivable empirical outcome, it lacks testability and, consequently, cannot be considered scientific in the strict sense (Popper, 1959). While this principle exerted a profound influence on 20th-century science, later philosophers such as Kuhn (1962) and Lakatos (1980) refined Popper’s ideas, emphasizing that theories evolve within broader research programs and historical paradigms rather than through simple cumulative falsification. Moreover, in response to the challenge posed by classical induction (i.e., the justification of generalizations derived from repeated observations), certain authors have proposed an approach known as *eliminative induction* (Popper, 1959; Jaynes, 2003; Caticha, 2008). This methodological approach entails not seeking confirmation of a hypothesis but, rather, proceeding by systematically excluding rival hypotheses that fail to align with the data. This principle, as elucidated by Caticha (2008), presents a coherent and enhanced interpretation of Karl Popper’s scientific methodology. According to Caticha (2008), eliminative induction introduces an intriguing variation to Popper’s scientific approach. While Popper posits that scientific theories can never be definitively proven true, only falsified, eliminative induction harmonizes seamlessly with these ideas. However, it shifts the focus: rather than fixating on the failure of falsification, the emphasis is directed toward success, i.e., the successful falsification of all rival theories that support the prevailing one (Caticha, 2008).

One promising avenue for formalizing this scientific strategy of model selection under uncertainty is provided by Bayesian inference. Far from being merely a method for parameter estimation, the Bayesian framework offers a general logic for comparing competing hypotheses in light of new evidence (Jaynes, 2003; Howson and Urbach, 2006). In this context, Bayesian inference may serve as an operational formalization of this scheme (Jaynes, 2003). Models are continually updated based on evidence, and those that yield less favorable predictions are progressively discarded in favor of those that, at the present moment, most effectively withstand the scrutiny of data. This approach aligns with falsificationism, yet it offers greater flexibility and a gradual progression. Notably, it is not predicated on classical induction. From this viewpoint, scientific advancement does not occur through confirmation or definitive proof, but rather through a logical process of eliminative induction that ascribes greater plausibility to models that endure critical evaluation while simultaneously maintaining the possibility of revision (Dienes, 2011).

In this article, we propose a critical examination of the primary etiological theories of AD in light of the aforementioned epistemological criteria. For each main theory, we will evaluate its falsifiability, the predictions it entails, the types of data that could undermine it, and its response to negative results. Our goal is not to discredit individual theories but to foster broader reflection on how knowledge in Alzheimer’s research is produced, evaluated, and sustained. We argue that many of the prevailing theories, despite their sophisticated biological underpinnings, lack epistemological informativeness. Consequently, we propose a methodological shift inspired by eliminative induction as a more productive and transparent approach for guiding future research.

## 2 Scientific theories and the problem of falsifiability

Karl Popper’s most influential contribution to 20th-century philosophy of science was the concept that a theory is scientific only if it is potentially falsifiable. This notion stands in contrast to the traditional inductive approach, which sought to establish general laws based on a multitude of concordant observations. Popperian falsificationism, on the other hand, reverses this approach. No amount of confirmation can ever render a theory true, while a single piece of contrary evidence, at least in principle, should be sufficient to refute it. Consequently, science does not progress through the accumulation of evidence, but rather by the elimination of hypotheses that fail empirical testing (Popper, 1959).

However, as Lakatos (1980) observed, the notion of falsifiability is more problematic than it may seem. Scientific theories rarely stand alone: they are embedded within broader research programs that include auxiliary hypotheses, methodological conventions, and statistical models. When predictions fail, it is often unclear whether the error lies in the core theory or in some peripheral assumption—a dilemma known as the Duhem–Quine problem. To address this ambiguity, Lakatos (1980) proposed a more nuanced framework distinguishing between the relatively stable “theoretical core” of a research program and its “auxiliary hypotheses,” which can be adjusted or replaced to accommodate new empirical findings. According to this view, a scientific program is not discarded at the first sign of falsification but can continue to evolve as long as its core remains intact and the adjustments concern only its auxiliary components. In a complementary vein, Kuhn (1962) emphasized that scientific progress rarely occurs through the straightforward rejection of theories. Periods of “normal science” are marked by the persistence of dominant paradigms despite accumulating anomalies, which are often reinterpreted or temporarily ignored. Paradigm shifts, therefore, do not result from logical falsification but emerge through complex historical and social processes that reshape the scientific consensus. Together, these reformulations highlight the dynamic and historically contingent nature of scientific inquiry, moving beyond Popper’s model of strictly cumulative and falsification-driven progress.

In light of these challenges, several philosophers and scientists have proposed a more pragmatic and progressive alternative: eliminative induction. Rather than requiring decisive falsification, this approach involves the comparative evaluation of rival theories, progressively discarding those that perform worse in light of the evidence (Sober, 1981; Jaynes, 2003; Howson and Urbach, 2006). Theories are not overthrown by a single counterexample but, rather, gradually lose plausibility as they fail to account for new data. As Caticha (2008) argues, this approach is consistent with Popperian philosophy, yet it reframes the logic of scientific progress as a dynamic process of model comparison and refinement, rather than binary confirmation or refutation.

This concept seamlessly aligns with the Bayesian methodology for inference. Within the Bayesian framework, each theory can be conceptualized as a probabilistic model that generates predictions with inherent uncertainty (Costa et al., 2022). Empirical evidence does not yield a definitive binary judgment (true/false), but rather continuously modifies our confidence in various models (van de Schoot et al., 2021). Theories that exhibit satisfactory predictive performance gain credibility, while those that fail to do so diminish it. Consequently, science transforms into a process of data-driven elimination, wherein the primary objective of experimentation is not to refute a theory in absolute terms, but rather to foster progress through the gradual exclusion of alternative hypotheses that are less compatible with the observed facts (Jaynes, 2003; Dienes, 2011).

Within the realm of clinical neuroscience, particularly in Alzheimer’s research, this approach may demonstrate exceptional utility. Indeed, current etiological theories frequently exhibit intricate complexity and multi-level structures, rendering their direct translation into precise predictions challenging. The research landscape is predominantly characterized by highly theoretical programs, which persistently adapt to novel data in response to refutation. Consequently, an approach rooted in classical falsifiability may risk its ineffectiveness. Conversely, a framework grounded in eliminative induction and Bayesian inference can provide a rational framework for comparing, updating, and ultimately discarding competing models, without asserting definitive refutation. In the following sections, we apply this perspective by treating each major etiological theory of Alzheimer’s as a rival hypothesis subject to eliminative evaluation, assessing its testable predictions, empirical resistance, and continuing heuristic value for future research.

## 3 The main etiological theories of Alzheimer’s disease

Over the past three decades, numerous theories have been proposed to elucidate the etiology of AD. Some of these theories have gained prominence, significantly influencing research directions, diagnostic criteria, and therapeutic interventions. Conversely, others have assumed a more peripheral or emerging role, yet they are garnering increasing attention due to the limitations exhibited by conventional theories. In this section, our objective is not to provide an exhaustive review, but rather to identify and discuss the primary theoretical families that are currently active, in accordance with the epistemological criteria outlined previously: the explicit formulation of predications, resistance to comparative analysis with empirical data, and the willingness to undergo rational elimination.

### 3.1 The amyloid cascade hypothesis

First proposed by Hardy and Higgins (1992), the amyloid cascade hypothesis has served as the dominant conceptual framework in Alzheimer’s disease research for over three decades. According to this model, the extracellular accumulation of amyloid-*β* (Aβ) peptides, especially the Aβ40 and Aβ42 isoforms, is hypothesized to be the initiating event in a pathophysiological cascade that ultimately leads to neurodegeneration and dementia (Hardy and Higgins, 1992; Roberts et al., 1993; Selkoe, 2000; Ethell, 2010; Anastasio, 2011; Tam and Pasternak, 2012; Castellani et al., 2019; Paroni et al., 2019; Petrella et al., 2019).

Aβ peptides originate from the proteolytic cleavage of the amyloid precursor protein (APP), a transmembrane glycoprotein, by the sequential action of two enzymes: β-secretase (BACE1) and *γ*-secretase (Tam and Pasternak, 2012). This cleavage produces fragments of varying lengths, including Aβ38, Aβ40, and Aβ42, with the latter being more hydrophobic and aggregation-prone (Selkoe, 2000). Under normal physiological conditions, Aβ is efficiently cleared from the brain through enzymatic degradation and transport mechanisms. However, with aging or in the presence of genetic mutations in genes such as APP, PSEN1, and PSEN2, Aβ clearance becomes impaired, leading to its accumulation in both soluble and insoluble forms (Castellani et al., 2019; Paroni et al., 2019). A key pathological feature of AD, senile plaques, is composed of extracellular deposits of Aβ in several morphological forms, including diffuse, dense-cored, and neuritic plaques (Roberts et al., 1993). In addition to insoluble amyloid fibrils that make up plaques, Aβ also exists in soluble oligomeric forms that are believed to be particularly neurotoxic. These oligomers can interfere with synaptic transmission, plasticity, and network function even in early stages of the disease, before the appearance of overt neuropathological hallmarks (Ethell, 2010). The accumulation of Aβ peptides is hypothesized to trigger a cascade of downstream events, including hyperphosphorylation of tau protein with subsequent formation of neurofibrillary tangles, activation of microglia and astrocytes leading to chronic neuroinflammation, disruption of calcium homeostasis and mitochondrial function, and ultimately synaptic degeneration and neuronal cell death (Selkoe, 2000; Anastasio, 2011; Castellani et al., 2019).

The amyloid cascade hypothesis makes clear empirical predictions: individuals with elevated brain A*β* levels should display or eventually develop cognitive decline, and therapeutic interventions that reduce Aβ production or enhance its clearance should attenuate disease progression. However, despite initial enthusiasm, a growing number of anti-amyloid clinical trials have failed to demonstrate cognitive benefits, even when Aβ burden was significantly reduced (Paroni et al., 2019; Petrella et al., 2019). This growing discrepancy between biomarker modification and clinical outcome has led to increasing criticism of the model’s explanatory sufficiency (Schneider et al., 2014). Nevertheless, the amyloid cascade hypothesis continues to influence both the clinical and research landscape. Aβ biomarkers such as PET imaging and cerebrospinal fluid Aβ42 concentrations are routinely used in diagnosis, patient stratification, and trial design. Although the hypothesis remains a central organizing principle in the field, it is now regarded as incomplete and in need of integration with alternative or complementary mechanisms that better capture the multifactorial nature of AD (Dominguez-Gortaire et al., 2025).

### 3.2 The tau proteinopathy hypothesis

The tau proteinopathy hypothesis proposes that the primary driver of AD pathology is not amyloid accumulation, but rather the abnormal modification and aggregation of the tau protein, a microtubule-associated protein involved in cytoskeletal stability and axonal transport (Maccioni et al., 2010; Kametani and Hasegawa, 2018; Arnsten et al., 2021; Chen and Yu, 2023). In physiological conditions, tau supports microtubule assembly and neuron structure, particularly in axons. However, in AD and other tauopathies, tau becomes hyperphosphorylated, misfolded, and prone to aggregate into paired helical filaments (PHFs) and eventually neurofibrillary tangles (NFTs), one of the histopathological hallmarks of the disease (Maccioni et al., 2010; Kametani and Hasegawa, 2018; Chen and Yu, 2023).

Unlike amyloid plaques, which often show weak correlation with symptom severity, neurofibrillary tangles strongly correlate with cognitive impairment and disease progression. This observation, initially documented by Braak and Braak (1991), revealed that the topographical distribution and density of tau pathology follow a consistent and hierarchical pattern, starting in the transentorhinal cortex and progressing through the hippocampus, limbic system, and neocortical regions. This staging system forms the basis of many neuropathological diagnostic criteria and supports the idea that tau burden is a more accurate proxy of clinical severity than amyloid load (Arnsten et al., 2021). It is important to note, however, that tau pathology in AD—which almost invariably co-occurs with amyloid-*β* deposition—may differ substantially from that observed in Primary Age-Related Tauopathy (PART), a common age-associated condition characterized by tau aggregation in the absence of amyloid (Nelson et al., 2009; Mungas et al., 2014).

According to this hypothesis, amyloid may play a permissive or triggering role, facilitating tau hyperphosphorylation and spread, but it is not the fundamental cause of neuronal death or cognitive decline. Some variants of the tau hypothesis interpret amyloid as a non-essential epiphenomenon, or at most a modulator of tau pathology, rather than the primary driver. Experimental data from transgenic mouse models and postmortem human studies have further shown that tau pathology alone can induce neurodegeneration in the absence of amyloid, whereas amyloid without tau does not necessarily lead to clinical symptoms (Maccioni et al., 2010; Arnsten et al., 2021).

In this context, therapeutic strategies targeting tau, such as inhibitors of tau kinases, anti-tau antibodies, or agents blocking tau aggregation and propagation, have gained increasing attention, particularly following the limited success of amyloid-based therapies (Congdon and Sigurdsson, 2018). Moreover, tau imaging via PET tracers has shown promise as a biomarker of disease stage and progression (Chen et al., 2021).

### 3.3 The inflammatory hypothesis

A growing body of research emphasizes the pivotal role of chronic neuroinflammation in AD, particularly the maladaptive activation of microglia in response to extracellular protein aggregates such as amyloid-*β* and tau (de Oliveira et al., 2021; Bowirrat, 2022; Heneka et al., 2025). Under physiological conditions, microglia act as the brain’s resident immune cells, clearing debris and modulating synaptic pruning. However, in AD, they often adopt a sustained pro-inflammatory phenotype that fails to resolve the underlying pathology and instead promotes neurodegeneration (Giri et al., 2024; Heneka et al., 2025).

One key genetic contributor to this process is the Triggering Receptor Expressed on Myeloid cells 2 (TREM2) gene, which encodes a receptor involved in microglial activation and phagocytosis. Rare variants in TREM2—such as the R47H mutation—have been associated with a 2- to 4-fold increased risk of developing late-onset AD, comparable in magnitude to the well-known APOE ε4 risk allele (Hou et al., 2022; Li et al., 2023). These mutations impair microglial ability to cluster around amyloid plaques, reducing clearance and exacerbating the inflammatory environment (Pascoal et al., 2021). Furthermore, persistent activation of glial cells—particularly microglia and astrocytes—leads to the chronic release of cytokines (e.g., IL-1β, TNF-*α*), reactive oxygen species, and complement factors, which in turn contribute to synaptic dysfunction, neuronal loss, and blood–brain barrier disruption (Boyd et al., 2022; Heneka et al., 2025). This cascade, while triggered by misfolded proteins, becomes self-perpetuating and may outlast the original insult, suggesting that neuroinflammation is not merely a secondary phenomenon but a key driver of disease progression.

## 4 Are theories falsifiable?

In the context of AD, the criterion of falsifiability is paramount for distinguishing between scientific explanations and *post-hoc* narratives. However, as previously discussed, falsifiability should not be interpreted rigidly and in a binary manner, but rather as an integral aspect of a systematic process of model selection. In this section, we comprehensively analyze each of the prominent theories presented, examining them through three fundamental questions: What specific predictions does it explicitly make? What empirical evidence would serve as a means of refuting its validity? Furthermore, to what extent has this theory undergone critical evaluation and comparative analysis with alternative models?

**Figure 1:**
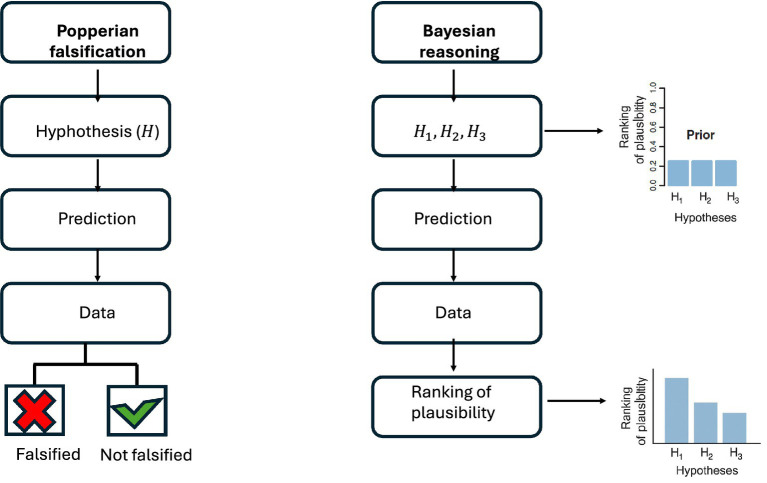
Two ways of evaluating scientific theories in Alzheimer’s disease. On the left, the Popperian binary model of falsification (a hypothesis is either falsified or survives). On the right, the Bayesian/eliminative approach, in which multiple hypotheses are compared, each receiving a degree of support from the data, leading to a ranking of plausibility.

As illustrated in Figure 1, a schematic comparison between Popperian falsification and Bayesian/eliminative reasoning clarifies these issues.

### 4.1 The amyloid cascade hypothesis

The amyloid hypothesis posits that the accumulation of β-amyloid peptide precedes and causes cognitive decline, while its removal either slows or prevents the progression of AD. In theory, this hypothesis is falsifiable. It would suffice to observe subjects with high amyloid load but no symptoms or patients with severe symptoms but no significant accumulation to challenge it. This is precisely what has been repeatedly observed (Aizenstein et al., 2008; Panza et al., 2019; Costa et al., 2023). For example, in the Mayo Clinic Study of Aging, >60% of individuals over 85 exhibited significant Aβ accumulation, yet <10% developed dementia (Roberts et al., 2008). However, the theoretical response to these anomalies has been the introduction of variants and sub-theories, such as the toxic oligomer hypothesis or long temporal models (Benilova et al., 2012; Stancu et al., 2014), which tend to preserve the central theory from invalidation. Moreover, almost all drugs designed to reduce amyloid have proven ineffective in significantly modifying the clinical course of the disease (Panza et al., 2019; Costa and Cauda, 2022; Costa et al., 2023).

Consequently, the amyloid cascade hypothesis has undergone multiple reformulations in response to contradictory or null findings. Instead of being abandoned or decisively revised in light of anomalies—such as the lack of clinical benefit following successful amyloid clearance (Mullane and Williams, 2013; Panza et al., 2019), or the presence of significant amyloid burden in cognitively intact individuals (Aizenstein et al., 2008)—the theory has been adapted by incorporating new auxiliary assumptions. These include the toxic oligomer hypothesis (Benilova et al., 2012), long preclinical latency models (Jack et al., 2018), and multi-factorial accounts involving tau, neuroinflammation, and vascular contributions (Heneka et al., 2015).

While scientific theories must evolve to remain viable, there is a critical point at which continuous accommodation of conflicting data risks undermining the theory’s operational falsifiability. As Popper (1959) emphasized, the strength of a scientific theory lies in its ability to make bold predictions that risk empirical refutation. When negative results are consistently absorbed through post hoc revisions, the theory becomes immune to decisive testing and, in Lakatos’ terms, shifts from a progressive to a degenerative research program (Lakatos, 1980). From the standpoint of eliminative induction (Jaynes, 2003; Caticha, 2008), this theoretical elasticity is epistemologically counterproductive. Scientific inference proceeds by comparing competing models and discarding those that fail to account for the data in a parsimonious and predictive manner. Although the amyloid hypothesis has not been formally falsified, its relative plausibility has declined sharply in light of mounting empirical anomalies and the increasing explanatory strength of alternative frameworks, such as tau pathology or neuroinflammatory cascades (Maccioni et al., 2010; Kametani and Hasegawa, 2018).

In this context, the theory no longer functions as a scientific discriminator. Its continued survival appears less due to empirical success and more to its adaptive flexibility, which—paradoxically—renders it less scientifically informative. As Dienes (2011) has argued, a theory that predicts everything in vague terms effectively predicts nothing, and its evidential weight is thereby diminished.

### 4.2 The tau proteinopathy hypothesis

The tau theory posits a correlation between the quantity and distribution of hyperphosphorylated tau aggregates and cognitive decline, with tau-PET metrics strongly predicting clinical trajectories in AD (Biel et al., 2022; Ioannou et al., 2025). Unlike amyloid, tau burden correlates more closely with symptom severity (Rabinovici et al., 2025).

Nevertheless, several critical challenges remain. Tau pathology occurs in cognitively unimpaired individuals, as exemplified by Primary Age-Related Tauopathy (PART; Crary et al., 2014; Chung et al., 2021), and is also present in other neurodegenerative diseases, including progressive supranuclear palsy and corticobasal degeneration, raising questions about its specificity for AD. Additionally, tau often co-localizes and interacts with TDP-43 pathology, particularly in LATE-NC and other AD cases (Nelson et al., 2019; Tomé et al., 2020; Youssef et al., 2025), complicating the attribution of neurodegeneration solely to tau. Attempts at anti-tau interventions, such as tau-targeting antibodies, have yet to yield conclusive clinical benefits (Singleton et al., 2025).

The causal relationship between tau and neurodegeneration remains only partially established, and the theory, while falsifiable, has not been rigorously tested against well-defined alternative models. Consequently, although tau provides substantial explanatory value, its predictive utility is limited, and it accommodates anomalies without formal refutation.

### 4.3 The inflammatory hypothesis

While this theory is supported by a growing body of molecular, genetic, and neuroimaging data, it still lacks a rigorous operational definition (Cantero-Fortiz and Boada, 2024). It remains unclear whether neuroinflammation is a cause, effect, or cofactor of neurodegeneration. Furthermore, its predictions are highly vague and diffuse: inflammation is present in numerous neurological conditions and is not specific to AD (Boyd et al., 2022; Giri et al., 2024). In this regard, the hypothesis possesses significant explanatory potential but still exhibits limited predictive or eliminative power.

The falsifiability of the neuroinflammatory hypothesis is constrained by the ambiguity of its predictions and the difficulty of isolating inflammatory effects from other concomitant pathogenic processes. As a result, the model accommodates multiple explanations that can fit almost any clinical profile. To render it truly testable, it would be imperative to formulate differential predictions, such as observing cognitive decline in the absence of amyloid or tau accumulations if inflammation were the primary cause—but such observations have not yet been unequivocally demonstrated. Presently, the model is theoretically falsifiable, but remains too flexible for rigorous eliminative selection.

## 5 Why falsifiability fails in Alzheimer’s clinical research

Although the criterion of falsifiability represents a methodological ideal in science, its practical application in Alzheimer’s research encounters profound and enduring challenges. As previously observed, the proposed main theories are, at the very least, amenable to testing. However, in both scientific and clinical contexts, even hypotheses that have amassed a substantial number of unfavorable data persist in being defended, refined, and supported. This discrepancy between epistemological theory and actual scientific practice raises pertinent questions. In this section, we examine four fundamental reasons that elucidate why falsifiability fails, or rather, is systematically circumvented, within the realm of AD research.

### 5.1 Biological and clinical complexity

AD is not a singular, causal, or homogeneous condition. Neurobiological and clinical evidence point toward a heterogeneous mosaic of mechanisms, progression patterns, and clinical manifestations. Some patients exhibit amyloid-dominant characteristics, while others display tau-dominant features (Mohanty et al., 2023). Others have vascular, metabolic, or inflammatory comorbidities, and factors such as age, genetics, pre-existing health conditions, and lifestyle significantly contribute to interindividual variability (Habes et al., 2020; Duara and Barker, 2022). Clinical syndromes of early-onset AD—including amnestic, visuospatial, and behavioral variants—further highlight the heterogeneity of Alzheimer’s presentations (Sirkis et al., 2022). The heterogeneous and multifaceted nature of the disease underscores the limitations of a unitary model and invites a more nuanced, stratified approach to understanding and treating AD (Jellinger, 2022; Avelar-Pereira et al., 2023).

The inherent complexity of this domain presents a challenge in formulating clear and unequivocal predictions. A theory can be salvaged by attributing the discrepancy between prediction and data to an atypical subpopulation, an alternative clinical progression, or a compensatory mechanism. However, in the absence of a formal model capable of integrating all these variables, the risk arises that any theory becomes adaptable to any evidence, thereby diminishing its effective falsifiability. This is not a deliberate flaw, but a pragmatic necessity: within a highly intricate domain, resistance to simplification and the proliferation of compatible explanations tend to reinforce models that provide extensive explanations but lack predictive power.

### 5.2 Lack of unique markers

In the field of AD, there are currently no completely specific and sensitive biomarkers that allow the dominant causal mechanism in each patient to be established with certainty. Currently available biomarkers—such as Aβ, tau, neurofilaments, metabolic imaging, or inflammatory markers—provide important data, but they are often overlapping, correlational, or interpretable in different ways depending on the model adopted (Galasko and Golde, 2013; Ferreira et al., 2014; Heneka et al., 2015, 2025; Jack et al., 2018). This ambiguity hinders the development of pivotal tests capable of distinguishing between competing theories. If a high biomarker can be interpreted as either a cause, an effect, or a compensatory epiphenomenon, the theory’s validity remains intact. Moreover, the absence of an unambiguous “biological signature” precludes the construction of robust eliminative experiments, which are fundamental to the rational scientific method.

### 5.3 Institutional resistance to paradigm shifts

As noted by Kuhn (1962), scientific revolutions do not arise from the mere refutation of a theory, but rather from its replacement by an alternative paradigm that better accounts for the available evidence. In the field of AD, the persistence of prevailing theories may plausibly reflect a combination of structural, institutional, and cultural factors rather than purely empirical success. These frameworks have enjoyed considerable structural and institutional advantages for decades, including preferential funding, publication bias, and regulatory alignment (Mullane and Williams, 2013; Panza et al., 2019). As a result, even in the face of repeated negative or inconclusive results, they have managed to maintain their dominant status. This phenomenon may be attributed to several institutional factors: funding agencies that continue to favor projects consistent with the traditional hypothesis; clinical guidelines that incorporate both amyloid and tau biomarkers into diagnostic criteria; and journals that exhibit a publication bias toward “positive” results aligned with the dominant paradigm. Within this context, falsifiability is not only compromised by epistemic considerations, but also by systemic inertia that safeguards accepted theories from eliminative pressures.

### 5.4 Academic incentives for theoretical preservation

The academic system often tends to emphasize productivity, continuity, and the ability to secure funding over the willingness to challenge one’s own hypotheses. Within such a context, researchers who have built their careers around a specific theory—particularly in a high-profile field such as Alzheimer’s—may be understandably inclined to defend it even in the presence of problematic data. Consequently, a dynamic of “defensive adaptation” of theories may plausibly emerge: instead of being abandoned, they are reformulated, articulated into sub-theories, enriched with latent variables, or transformed into multifactorial frameworks that are less easily falsifiable. This mechanism could be viewed as analogous to what Lakatos (1980) described as a “degenerative research program,” in which the central theory is progressively safeguarded by a defensive ring of auxiliary assumptions that plausibly impede its eliminative replacement.

## 6 Toward a new epistemology for Alzheimer’s disease

In light of the systemic difficulties encountered by falsifiability as an operational criterion in Alzheimer’s research, we argue that it is increasingly urgent to adopt an alternative epistemological view that respects the complexity of the phenomenon while providing rational tools for evaluating and advancing theories.

A promising proposal is to shift the focus from attempting to falsify individual hypotheses to comparatively evaluating their relative plausibility based on empirical evidence. This is the heart of Bayesian inference, which interprets theories as probabilistic models to which degrees of credibility can be assigned, updated, and compared. In this perspective, science does not advance through the definitive verification or refutation of hypotheses, but through a continuous process of progressive elimination of the least plausible models, consistent with the idea of eliminative induction (Popper, 1959; Forster and Sober, 1994; Jaynes, 2003; Caticha, 2008).

Theories that predict empirical data well obtain greater quantitative support than those that predict them less accurately, without any claim to absolute truth. This comparative rationality, which reflects Popper’s principle of comparison between rival theories, is formalized in the Bayesian framework through the Bayes Factor (BF; Kass and Raftery, 1995). The BF is an index that measures how much more likely the observation of data is under one theory than under another (Jeffrey, 1948; Kass and Raftery, 1995). If $BF_{10}=10$ , for example, it means that the data are 10 times more compatible with model 1 than with model 0. This approach allows us to quantify the strength of evidence in favor of a hypothesis, promoting transparent and replicable decisions. Furthermore, as Dienes (2011) pointed out, the BF is a quantitative tool for rigorously testing theories, in line with the spirit of falsificationism, but without relying on arbitrary thresholds such as *p*-values or binary acceptance/rejection logic.

A concrete example of the application of this approach in the context of AD clinical research is provided by Costa and Cauda (2022), who conducted a Bayesian reanalysis of phase III of the clinical trial of aducanumab, a highly controversial anti-amyloid drug. This study used the BF to directly compare the empirical support for the drug’s efficacy against the null hypothesis. The results showed that, unlike the traditional interpretation based on frequentist tests, the evidence in favor of efficacy was weak or even contrary. Beyond re-analyses of clinical trials, Bayesian disease-progression modeling has been applied to cognitive, functional, biomarker, and imaging data coming from the Alzheimer’s Disease Neuroimaging Initiative to characterize long-term disease dynamics. Specifically, Li et al. (2018) developed a latent-time joint mixed-effects model that captures individual variability, quantifies uncertainty, and improves predictive accuracy across disease stages. Bilgel and Jedynak (2019) proposed a probabilistic template of disease progression to predict time to dementia, explicitly propagating uncertainty and enabling clinically actionable forecasts. Taken together, these applications show that Bayesian inference serves not only as an inferential tool but also as a conceptual bridge between theory, data, and decision-making.

It is essential to recognize that, within this framework, models are no longer regarded as ‘true’ representations of biological reality, but rather as operational tools for organizing, synthesizing, and predicting data (Costa et al., 2022). Their value depends on their ability to adapt to evidence without overfitting, to generate specific predictions, and to be explicitly compared with alternative models. Accordingly, a transformation in the way Alzheimer’s research is conducted becomes desirable: a more comparative science, which places theories in explicit competition; more predictive, which makes empirical expectations explicit in advance; and more transparent, which values negative outcomes as an integral part of scientific progress. In conclusion, if falsifiability proves inadequate as a guiding criterion for Alzheimer’s science, Bayesian inference offers an alternative and more operational path: a form of eliminative inductive scientific rationality capable of guiding research in a manner more consistent with the complexity of the phenomenon and the challenges of contemporary medicine.

## 7 (Ad interim) conclusion

Currently, Alzheimer’s disease research is at a critical juncture, not primarily due to a scarcity of data, but rather to a latent epistemological crisis. This crisis manifests in the form of an abundance of theories, limited explicitly comparative models, numerous adaptable predictions, and a scarcity of truly discriminating experiments. While the criterion of falsifiability, although noble and central to the philosophy of science, has proven ineffective in providing an operational framework for the rational selection of theories in the context of AD, its practical inapplicability does not stem from a transgression of scientific principles. We hypothesize that this situation may arise from a confluence of structural factors, including the biological and clinical complexity of the phenomenon, the absence of unequivocal markers, institutional resistance to change, and academic incentives that favor the preservation of dominant hypotheses. In this scenario, the primary concern is not to identify the definitive theory, but rather to reformulate the research questions. Instead of focusing on the causes of Alzheimer’s, we should shift our focus to identifying models that best predict the observed data, considering various subpopulations, degrees of uncertainty, and alternative explanations. This transformation aligns with a scientific approach that emphasizes operationality, comparability, and decision-making under uncertainty conditions (Jaynes, 2003; O’Hagan and Forster, 2004; Howson and Urbach, 2006; Caticha, 2008).

Bayesian inference serves as a natural framework for this transformation. It does not assert the definitive confirmation or falsification of theories, but rather facilitates their updating based on evidence and progressive selection of the most compatible models with the data. In this sense, it embodies a form of scientific rationality that draws upon Popper’s intuition of comparing conjectures, yet enhances its robustness and applicability in intricate contexts. The Bayesian approach, by its inherent nature, employs eliminative induction, progressively discarding the least plausible models without presuming the ultimate truth has been discovered (Jaynes, 2003). The implications of this shift in perspective may be profound. In research practice, comparative protocols should be encouraged, formal models capable of generating specific predictions developed, and negative findings explicitly valued as essential evidence. Clinically, it becomes crucial to recognize disease heterogeneity and to adopt stratified, probabilistic approaches to diagnosis and treatment that weigh the relative plausibility of different pathogenic mechanisms in each patient.

Ultimately, addressing AD necessitates not only novel theories but also a novel epistemology. A science that is more circumspect in its assertions but more rigorous in its methodologies; a science that is more receptive to a multitude of models yet more demanding in its assessment of their efficacy. Only then can we transform the current theoretical impasse into a tangible opportunity for advancement.

## Data Availability

The original contributions presented in the study are included in the article/supplementary material, further inquiries can be directed to the corresponding author.
